# Developing an artificial intelligence method for screening hepatotoxic compounds in traditional Chinese medicine and Western medicine combination

**DOI:** 10.1186/s13020-022-00617-4

**Published:** 2022-05-17

**Authors:** Zhao Chen, Mengzhu Zhao, Liangzhen You, Rui Zheng, Yin Jiang, Xiaoyu Zhang, Ruijin Qiu, Yang Sun, Haie Pan, Tianmai He, Xuxu Wei, Zhineng Chen, Chen Zhao, Hongcai Shang

**Affiliations:** 1 Key Laboratory of Chinese Internal Medicine of Ministry of Education, Dongzhimen Hospital, Beijing University of Chinese Medicine, Beijing, China; 2 School of Computer Science, Fudan University, Shanghai, China; 3 Present Address: Institute of Basic Research in Clinical Medicine, China Academy of Chinese Medical Sciences, Beijing, China

**Keywords:** AI, DILI, Machine learning, Deep learning, TCM-WMC, Safety assessment

## Abstract

**Backgrounds:**

Traditional Chinese medicine and Western medicine combination (TCM-WMC) increased the complexity of compounds ingested.

**Objective:**

To develop a method for screening hepatotoxic compounds in TCM-WMC based on chemical structures using artificial intelligence (AI) methods.

**Methods:**

Drug-induced liver injury (DILI) data was collected from the public databases and published literatures. The total dataset formed by DILI data was randomly divided into training set and test set at a ratio of 3:1 approximately. Machine learning models of SGD (Stochastic Gradient Descent), kNN (k-Nearest Neighbor), SVM (Support Vector Machine), NB (Naive Bayes), DT (Decision Tree), RF (Random Forest), ANN (Artificial Neural Network), AdaBoost, LR (Logistic Regression) and one deep learning model (deep belief network, DBN) were adopted to construct models for screening hepatotoxic compounds.

**Result:**

Dataset of 2035 hepatotoxic compounds was collected in this research, in which 1505 compounds were as training set and 530 compounds were as test set. Results showed that RF obtained 0.838 of classification accuracy (CA), 0.827 of F1-score, 0.832 of Precision, 0.838 of Recall, 0.814 of area under the curve (AUC) on the training set and 0.767 of CA, 0.731 of F1, 0.739 of Precision, 0.767 of Recall, 0.739 of AUC on the test set, which was better than other eight machine learning methods. The DBN obtained 82.2% accuracy on the test set, which was higher than any other machine learning models on the test set.

**Conclusion:**

The DILI AI models were expected to effectively screen hepatotoxic compounds in TCM-WMC.

## Introduction

Drug-induced liver injury (DILI), one of the commonest and serious adverse drug reactions, is the dominant cause for terminating clinical trials or withdrawing new drugs [1]. Statistical analysis showed that 15 of the 47 withdrawn drugs were due to hepatotoxicity, accounting for 31.9% [2]. An observational cohort study showed that DILI was the main cause for acute liver injury (ALF), accounting for 11% in the USA between 1998 and 2013 [3]. The incidence rate of DILI is 23.8 per 100 thousand people in China [4], attracted more research attentions.

The pathogenesis of DILI is not completely clear. Many risk factors lead to DILI, such as the role of host genetic, immunologic, and metabolic factors as well as drug and environmental effects [5, 6]. Although many methods have been developed to predict and assess the risk of DILI, mature and highly accurate methods are still insufficient. Classic methods, including experiments in vitro and in vivo, played a crucial role in predicting DILI. However, drug combinations have become common as multiple diseases coexisting. TCM-WMC have also been increasingly recognized [7–9], covering almost all clinical therapeutic areas in China [10], which increased the complexity of compounds ingested. Hence, it’s essential to develop an effective method to screen hepatotoxicity of compounds in TCM-WMC.

With the development of new computing technologies, artificial intelligence (AI) models have been widely used in cheminformatics [11], medical imaging [12], diagnostics [13], bioinformatics [14], and other fields, provided new ideas for screening hepatotoxic compounds. Machine learning and deep learning methods have been increasingly applied to screen hepatotoxic compounds, which treat the high-dimensional chemical structure information as vectors and calculate for prediction or classification purpose in an efficient way [15–17]. In this study, DILI dataset is collected to establish AI models for screening hepatotoxic compounds in TCM-WMC.

## Methods

### Method design

DILI dataset was collected from public databases and published literatures. Nine machine learning models and a deep learning model were constructed with combined DILI dataset. A better performance model would be chosen to screen hepatotoxic compounds in TCM-WMC.

### DILI dataset collection

The compounds in DILI combined dataset were retrieved from the DILIrank [18], LiverTox [19], LTKB [20], Hepatox [21]. The annotations in DILIrank were assigned four different severity classes by considering DILI-related market withdrawals and warnings [18]. LiverTox contains comprehensive and evidence-based information on drug, dietary supplement, and herbal-induced liver injury [19]. Liver Toxicity Knowledge Base (LTKB) contains drugs whose potential to cause DILI in humans using the FDA-approved prescription drug labels [20]. Hepatox is a data base on the hepatotoxic drugs file published every year in Gastroentérologie Clinique et Biologique [21]. The keywords of "liver damage", "Drug-induced liver injury (DILI)”, “hepatotoxicity”, “liver toxicity”, “liver failure”, “liver injury”, “hepatitis”, “jaundice”, “cholestasis”, “liver protection”, “hepatoprotective”, “hepatoprotection”, “Herb-induced liver injury (HILI)" were searched in PubMed (https://www.ncbi. nlm.nih.gov/pubmed/), Nature(https://www.nature.com/), Science Online (http://www.sciencemag.org/), Elsevier Science Direct (https://www.Sciencedirect.com), Springer (https://link.springer.com/), Wiley (https://onlinelibrary.wiley.com/), Oxford Academic (https://academic.oup.com /journals/) and other publishers’ databases to search the relevant literatures with DILI dataset. The search time was limited to 1999–2021. Duplicates from different sources and compounds without structures were excluded.

### AI model construction

Chemical structures of compounds were coded with SMILES (simplified molecular input line entry system). PaDEL-Descriptor software [22] was used to calculate the molecular descriptor and fingerprint of each compound based on SMILES string. PaDEL-1D and 2D descriptors of all compounds were calculated using PaDEL-Descriptor software (Yap, 2011). PaDEL-1D and 2D contained 1444 descriptors, including atom type electrotopological state (Estate) descriptors, Crippen’s logP, and molecular linear free energy.

**Fig. 1:**
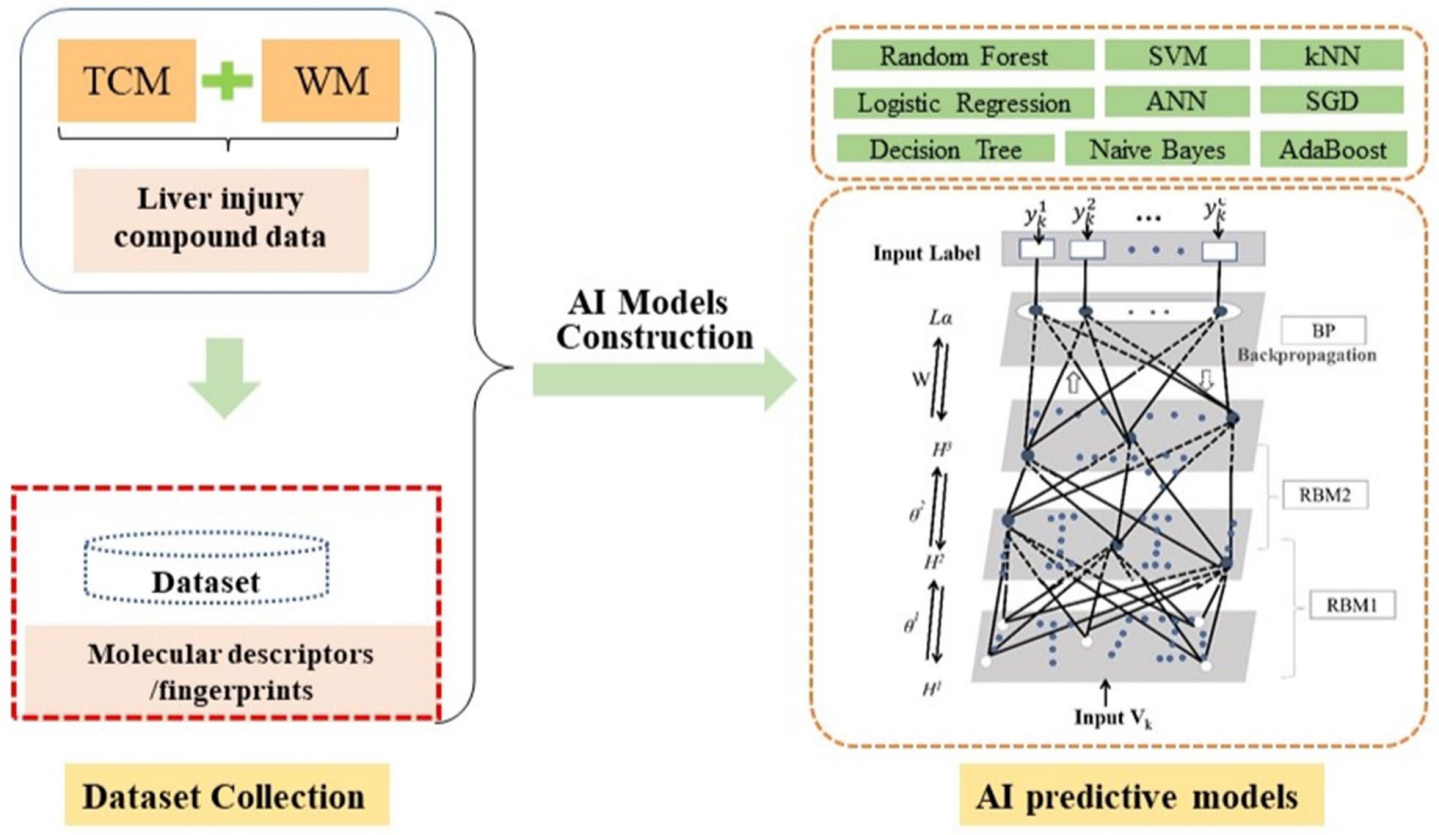
Workflow for the study of screening hepatotoxic compounds in TCM-WMC based AI methods. DILI Dataset was collected from public databases and published literatures. PaDEL was used to calculate molecular descriptors/fingerprints of DILI dataset compounds. Nine machine learning models of Stochastic gradient descent (SGD), k-nearest neighbor (kNN), Support vector machine (SVM), Naive bayes (NB), Decision tree (DT), Random forest (RF), Artificial neural network (ANN), Adaboost, Logistic regression (LR) and one deep learning model (DBN) were adopted to develop DILI AI models

The machine learning (ML) methods of SGD (Stochastic Gradient Descent), kNN (k-Nearest Neighbor), SVM (Support Vector Machine), NB (Naive Bayes), DT (Decision Tree), RF (Random Forest), ANN (Artificial Neural Network), Adaboost, LR (Logistic Regression) were adopted to build liver injury AI models. Two restricted Boltzmann machines (RBM) of deep belief network (DBN) were also constructed in this research. All these AI methods were trained on the same dataset, which was randomly divided into training set and test set at a ratio of 3:1 approximately. The workflow for the study of screening hepatotoxic compounds in TCM-WMC based AI methods was showed in Fig. 1.

### Statistics for model evaluation measures

Five important model evaluation measures for ML methods, including classification accuracy (Eq. 1), Precision (Eq. 2), Recall (Eq. 3), F1 score (Eq. 4), and area under the curve (AUC) of receiver-operating characteristic (ROC) were applied to assess the performance of each model. Therein, AUC represented the area under the ROC (Receiver operating characteristic) curve and the coordinate axis, CA represented the classification accuracy, Precision was how close the measured values that were to each other, Recall represented the recall rate. And the calculation formula of F1 score was as the Eqs. (4).

1 $$A{\text{cc}}uracy = \frac{TP + TN}{{TP + TN + FP + FN}}$$

2 $$P{\text{recision}} = \frac{TP}{{TP + FP}}$$

3 $$Se{\text{n}}sitivity = {\text{Re}} call = \frac{TP}{{TP + FN}}$$

4 $$F1 = \frac{{2 \times \Pr {\text{e}}cision \times {\text{Re}} call}}{{\Pr {\text{e}}cision + {\text{Re}} call}}$$

## Results

**Fig. 2:**
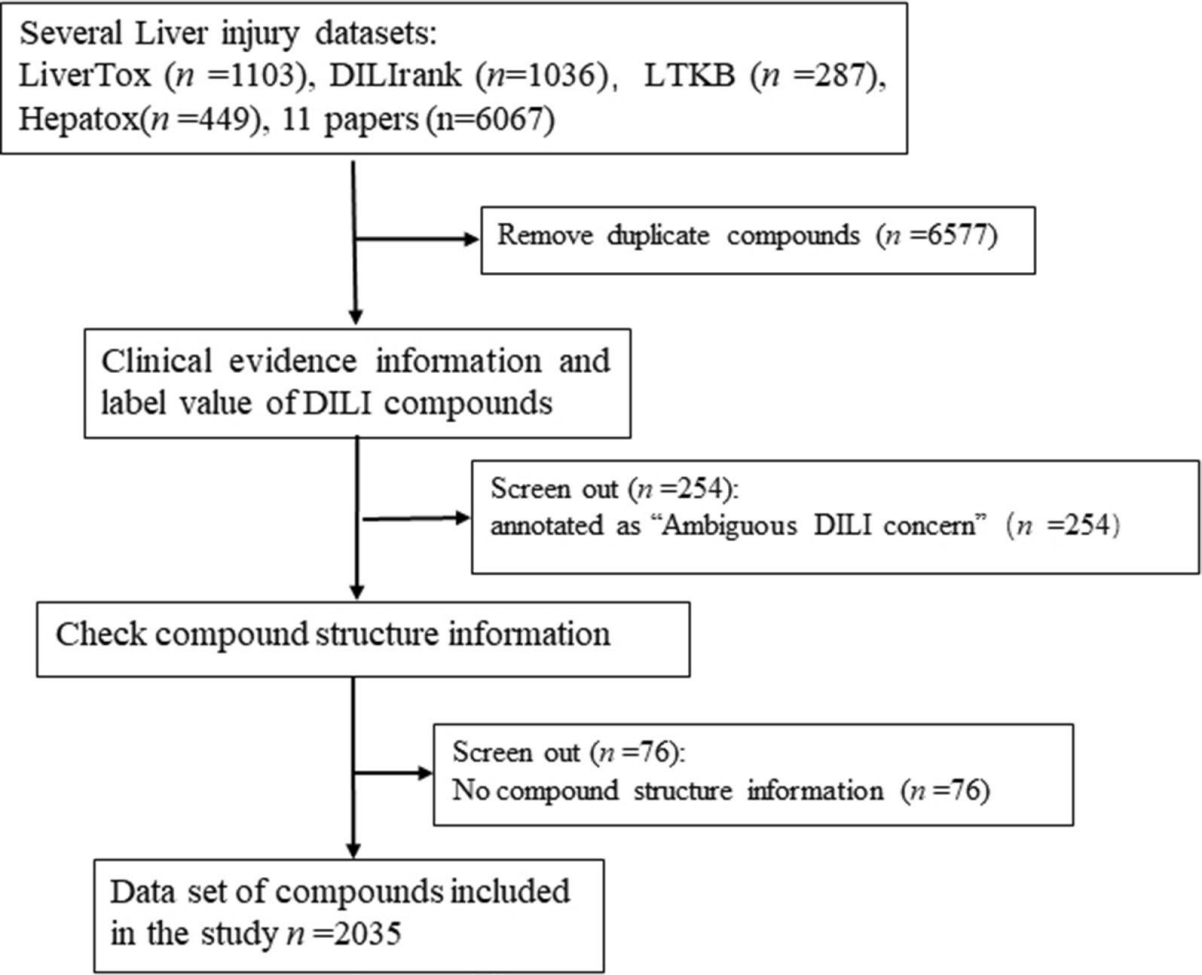
DILI dataset screening process

Two thousand eight hundred and seventy-five compounds were obtained from 4 DILI-related databases, and 6067 compounds were from 11 datasets in published DILI-related literatures [23–33]. After excluding the duplicate compounds, 2365 compounds was obtained. Subsequently, 254 drugs were also excluded annotated as “Ambiguous DILI concern” in DILIrank. Drugs without structure information were excluded after checking their structure information. At last, a total of 2035 liver injury compounds were collected in this research, as shown in Fig. 2.

In the total dataset, 1505 compounds were as the training set and 530 compounds were as the test set. The ratio of the number of liver injury and non-liver injury compounds in the training set was 1125:400, and the number ratio of liver injury and non-liver injury compounds in the test set was 380:130, respectively.

As listed in Table 1, CA ranged from 0.686 to 0.838, F1 ranged from 0.671 to 0.827, Precision ranged from 0.685 to 0.832, Recall ranged from 0.686 and 0.838, AUC ranged from 0.621 to 0.814 (Receiver-operating characteristic curve, ROC) for each model on the training set. CA ranged from 0.675 to 0.767, F1 ranged from 0.636 to 0.731, Precision ranged from 0.555 to 0.739, Recall ranged from 0.675 and 0.767, AUC ranged from 0.544 to 0.739 (ROC) for each model on the test set.

**Table 1:** AI models for drug-included liver injury

Dataset	ML method	AUC	CA	F1	Precision	Recall
Training set	SGD	0.647	0.709	0.715	0.723	0.709
	kNN	0.654	0.722	0.698	0.690	0.722
	SVM	0.785	0.795	0.791	0.788	0.795
	DT	0.710	0.756	0.760	0.764	0.756
	RF	0.814	0.838	0.827	0.832	0.838
	Adaboost	0.785	0.792	0.788	0.785	0.792
	ANN	0.621	0.737	0.671	0.685	0.737
	LR	0.746	0.776	0.757	0.757	0.761
	NB	0.632	0.686	0.694	0.711	0.686
Test set	SGD	0.627	0.682	0.694	0.712	0.682
	kNN	0.574	0.745	0.636	0.555	0.745
	SVM	0.669	0.747	0.712	0.710	0.747
	DT	0.544	0.680	0.679	0.678	0.680
	RF	0.739	0.767	0.731	0.739	0.767
	Adaboost	0.614	0.708	0.707	0.707	0.708
	ANN	0.647	0.694	0.696	0.697	0.694
	LR	0.656	0.733	0.694	0.688	0.733
	NB	0.598	0.675	0.648	0.705	0.675

*LR* Logistic regression, *RF* Random forest, *SVM* Support vector machine, *kNN* k-nearest neighbor, *DT* Decision tree, *NB* Naive bayes, *ANN* Artificial neural network, *SGD* Stochastic gradient descent

**Fig. 3:**
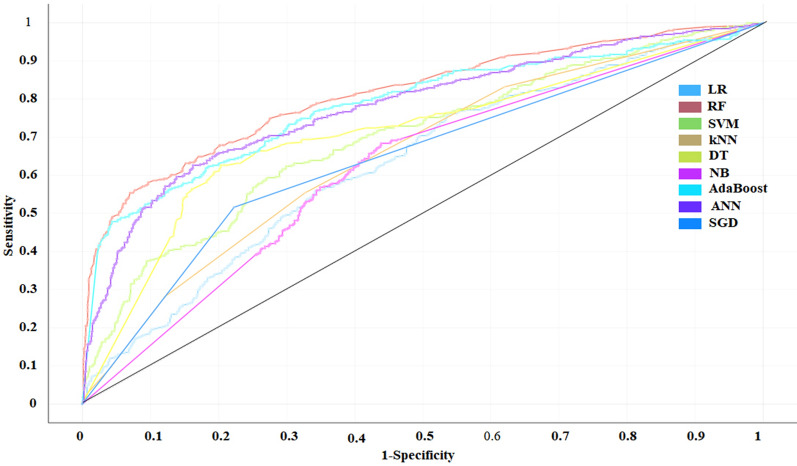
ROC curve of training set of DILI screening model based on AI model. LR: Logistic regression, RF: Random forest, SVM: Support vector machine, kNN: k-nearest neighbor, DT: Decision tree, NB: Naive bayes, AdaBoost, ANN: Artificial neural network, SGD: Stochastic gradient descent

**Fig. 4:**
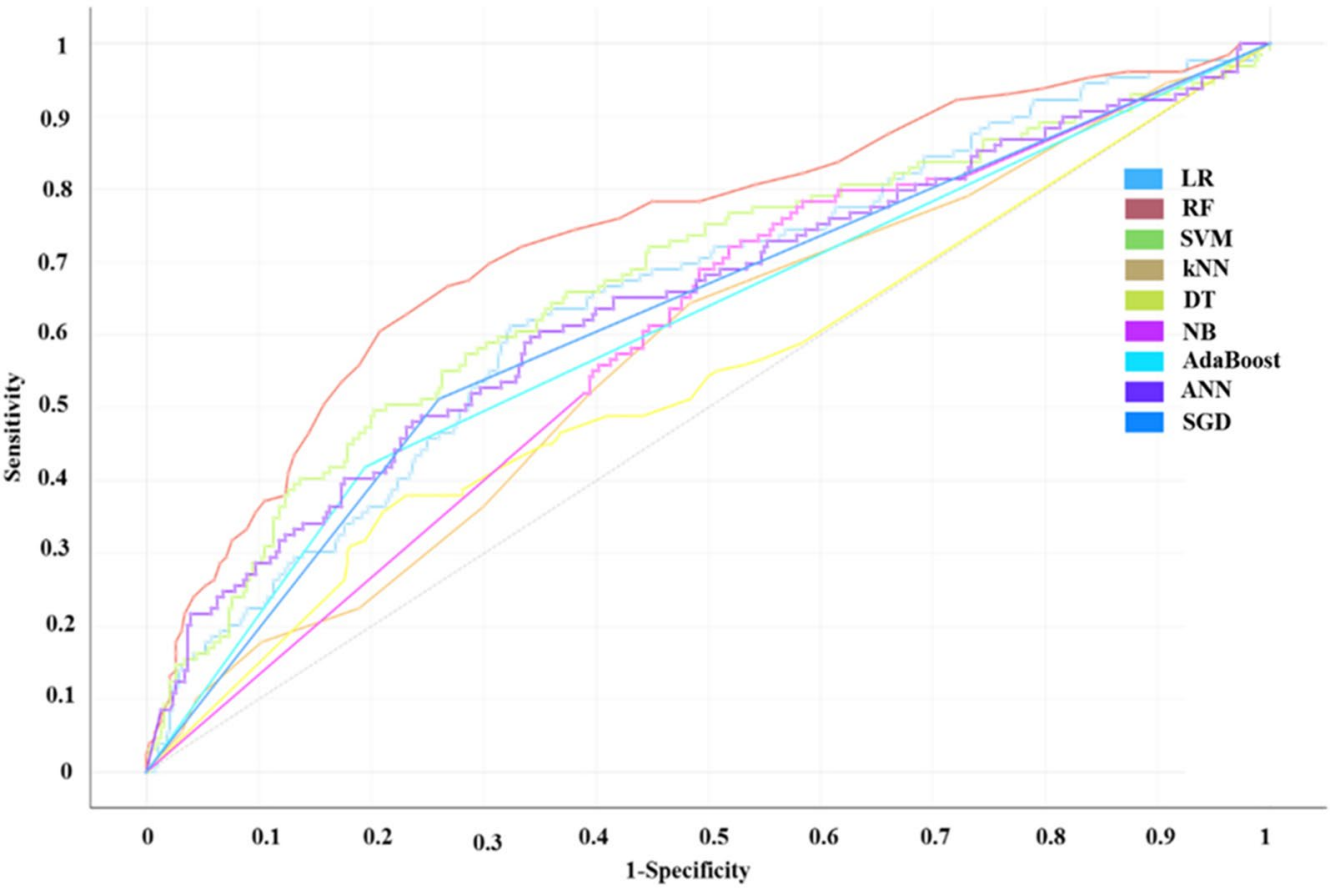
ROC curve of test set of DILI screening model based on AI model. LR: Logistic regression, RF: Random forest, SVM: Support vector machine, kNN: k-nearest neighbor, DT: Decision tree, NB: Naive bayes, AdaBoost, ANN: Artificial neural network, SGD: Stochastic gradient descent

Above model results showed that RF could achieved the best results than other machine learning methods on both the training set and the test set (Figs. 3 and 4). DBN obtained 82.2% accuracy on test set, which the number of hidden layers was 100, the batchsize was 25, and the learning rate was 1 with 400 iterations. This accuracy was higher than that of nine machine learning models on the test set.

## Discussion

In clinical practice, DILI lacks specific and sensitive diagnostic criteria, and the critical part of diagnosis depends on eliminating a series of diseases through blood testing. Once DILI events occur, the drug will be discontinued for the patient or even withdrawn from the market. Thus, it is urgent to develop a new approach to screen DILI.

Multiple compounds entered into the human body when TCM combined with WM. The complexity of the chemical compounds of TCM makes DILI extremely complicated in TCM-WMC, also increasing the risk of metabolic DILI. Early warning of compounds with hepatotoxicity has a vital clinical medication significance in TCM-WMC. Traditional DILI identification relies on animal experiments and clinical trials, risk factor assessment and case reports, etc. Above methods were not only inefficient but costly in terms of manpower, material, and financial resources. Besides, there may be a gap between experimental animals and mankind, reducing applicability of hepatotoxicity test results in humans. By comparison, recent DILI AI models were constructed based on mathematical models. For instance, quantitative structure–activity relationship (QSAR) model, one representative, was able to speculate on the specific physical, chemical and biological properties of compounds on the basis of structural information of known compounds, thereby achieving qualitative or quantitative screening of unknown compound.

It was challenging and crucial to systematically optimize the description form and their combinations of compounds. SMILES describes a three-dimensional chemical structure with a string of characters. Chemical structures can be characterized by a set of numerical values called molecular fingerprints or descriptors. These compound molecular descriptors or molecular fingerprints with high-dimensional information need to be processed with the application of information technologies, such as AI. Therefore, in this study, AI models was built for screening hepatotoxic compounds in TCM-WMC, with the 9 machine learning models (SGD, kNN, SVM, NB, DT, RF, ANN, Adaboost, LR) and one deep learning model (DBN). We found that DBN model had a better model performance than other 9 machine learning methods.

Basic machine learning models become progressively better at making predictions or decisions, which still need some guidance. More specifically, deep learning is considered to be the evolution of machine learning. It uses a programmable neural network to enable machines to make accurate decisions without humans help. DBN is composed of multi-layer unsupervised restricted Boltzmann machine (RBM) network and one layer supervised back propagation (BP) network.. The training process of DBN can be implemented from low to high level to train multilayer RBMs. Each RBM layer was trained by using the hidden unit (H) of the previous layer as the input/visible unit (V). The descriptors and fingerprints of drug or compounds were used as input V_*k,*_ and the binary classification with one layer supervised back propagation (BP) network of liver injury is used as input label. The combination of unsupervised RBM and supervised BP implements the supervised prediction of hepatotoxic compounds. After the training of the multi-layer RBMs, the liver injury of the prototype compounds/metabolic compounds of TCM-WMC is gradually categorized, to make a rapid warning for hepatotoxicity of compounds in TCM-WMC.

This study only focuses on the risk of liver injury caused by a single compound of TCM-WMC, and provides a path for the prediction of hepatotoxicity of more new compounds formed through the interaction between compounds from TCM and WM. In future research, mechanisms of liver injury in TCM-WMC needs to be further explained. We will further focus on the liver injury possible risk caused by TCM-WMC metabolites or interaction among complex TCM-WMC compounds by calculating more compounds data. Overall, this study will provide guidance for the safe utilization of TCM-WMC and improve the diagnosis and treatment ability of clinicians.

## Conclusion

We compared the DILI models’ performance of different machine learning and deep learning models, and found that DBN model had better model performance than other 9 machine learning methods. Therefore, this method of DBN for screening hepatotoxic compounds in TCM-WMC may be helpful to guide the clinical standards and safe medication, and avoid the risk of liver injury in the clinical combination.

## Data Availability

All data used in the presented study can get from the corresponding author upon request.

## Abbreviations

TCM-WMC: Traditional Chinese medicine and western medicine combination
DILI: Drug-induced liver injury
kNN: K-nearest neighbor
SVM: Support vector machine
NB: Naive bayes
DT: Decision tree
RF: Random forest
ANN: Artificial neural network
LR: Logistic regression
DBN: Deep belief network
CA: Classification accuracy
AUC: Area under the curve
AI: Artificial intelligence
ALF: Acute liver injury
ML: Machine learning
SGD: Stochastic gradient descent
ANNs: Artificial neural networks
RBM: Restricted boltzmann machine
ROC: Receiver operating characteristic
QSAR: Quantitative structure–activity relationship
